# Integration of Palliative Care Into Primary Health Care: Model of Care Experience

**DOI:** 10.7759/cureus.8866

**Published:** 2020-06-27

**Authors:** Sami A Alshammary, Balaji Punalvasal Duraisamy, Lobna Salem, Abdullah Altamimi

**Affiliations:** 1 Palliative Care, King Fahad Medical City, Riyadh, SAU; 2 Palliative Oncology, National Cancer Institute, Cairo University, Cairo, EGY; 3 Pediatric Emergency Medicine, King Fahad Medical City, Riyadh, SAU

**Keywords:** palliative, primary health care, model of care experience, integration of palliative care service

## Abstract

Background

The World Health Organization (WHO) has recognized access to palliative care as a basic human right. Palliative care service has been established in Saudi Arabia for more than two decades; however, it is still limited to secondary and tertiary healthcare institutions. While primary care is the first level of care in the Saudi healthcare system and covers the largest amount of the population, palliative care is still far from implemented at this level.

Objectives

This study sought to evaluate the outcome of integrating palliative care service at the primary healthcare level and assess patient satisfaction with services provided by primary healthcare centers (PHCCs).

Results

Two hundred patients participated in the study, including 50 new patients and 150 existing patients for follow-up. One hundred ten patients, in addition to 200 caregivers, attended the clinic. Twenty percent were on active oncology treatment. The no-show rate was 45%, and the overall satisfaction score was 90%.

Conclusion

Palliative care service integration into primary care is beneficial for improving access to early palliative care, and subsequently, improving symptom control, compliance with cancer treatment, quality of life, and overall satisfaction. This model will be implemented in all PHCCs in Saudi Arabia.

## Introduction

The prevalence of the chronic disease is rising worldwide. A World Health Organization (WHO) report from 2001 stated that chronic diseases were responsible for approximately 60% of the 56.5 million reported deaths, and cancer was among the leading health issues contributing profoundly to global morbidity and mortality [1]. Furthermore, the prevalence of non-communicable diseases and the number of aging populations are steadily rising [2,3]. All these factors together will add a significant burden on the healthcare system and healthcare providers, as well as contribute to poor healthcare system promotion around the world. In palliative care service, for example, it is estimated that only 14% of people in need of that service receive it [3]. Saudi society is not secluded from the international situation; in fact, the cancer prevalence rate in Saudi Arabia is similar to that of Western countries, which could be attributed to demographics and lifestyle of the population [2]. Therefore, the expansion of palliative care services to all levels of the healthcare system is imperative.

This study seeks to evaluate the outcome of integrating palliative care service at the primary healthcare level and assess patient satisfaction in relation to services provided by primary healthcare centers (PHCCs).

## Materials and methods

A cross-sectional study was conducted from March 22, 2018, to March 22, 2020. A total of 200 participants were randomly selected, and a patient experience and satisfaction survey was used. A PHCC in an outskirt district of Riyadh was identified. Family physicians who work at the PHCC were trained for three months on the basics of palliative care. The clinic operated every Thursday for five hours in the morning. Patients who lived nearby were referred to the palliative clinic. After each clinic visit, the patient or the caregiver was asked to fill out the patient experience and satisfaction survey. The survey included four questions with five answer options for each, ranging from extremely satisfied to extremely dissatisfied. The patient or caregiver responded to each question by selecting the answer that best represented his/her opinion, and the Model of Care (MOC) project officer collected all completed surveys. Inclusion criteria included all cancer survival patients. A family member or caregiver accompanied the patient. Exclusion criteria included non-cancer patients.

Statistical analysis

Descriptive statistics were used to analyze participants' sociodemographic data, including age and education. Frequencies and percentages were used. All statistical analyses were performed using Statistical Analysis System software, V9.1 (SAS Institute, Cary, NC).

## Results

 

One hundred ten participants responded to the survey. Approximately 45% of participants were women, and the average age was 56 years. Palliative patients comprised 70% of the participants and caregivers comprised 30%. The overall participant satisfaction rate was 88%. In response to the question about waiting time before the clinic, 58 participants (83%) were extremely satisfied. Ten participants (9%) were satisfied, and only one participant (1%) was dissatisfied with the waiting time at the clinic.

Regarding information on the purpose of the palliative clinic and whether the information given was appropriate, 98 participants (89%) were extremely satisfied, four participants (4%) were neutral, and none had complaints about the information provided. These results reflect the efficiency of the education provided at the primary healthcare level. In response to the question about respecting patient dignity during the clinical encounter, 106 participants (96%) were extremely satisfied with the way they had been treated by the primary healthcare staff at the clinic. No one provided any negative comments regarding this issue. For the overall palliative care clinic evaluation, 93 participants (84%) were extremely satisfied, three participants (3%) were satisfied, and one participant was dissatisfied. The overall participant satisfaction rate with the palliative care clinics at the PHCCs was 88% (Table 1).

**Table 1:** Palliative care: overall satisfaction in primary healthcare

No.	Question	Extremely Satisfied, n (%)	Satisfied, n (%)	Neutral, n (%)	Dissatisfied, n (%)	Extremely Dissatisfied, n (%)
1	Are you satisfied with the waiting time prior to consultation?	91 (83%)	10 (9%)	8 (7%)	0 (0%)	1 (1%)
2	Were you provided with sufficient information today to understand the purpose of and service being provided during the palliative care clinic?	98 (89%)	8 (7%)	4 (4%)	0 (0%)	0 (0%)
3	Were you treated with dignity and respect?	106 (96%)	3 (3%)	1 (1%)	0 (0%)	0 (0%)
4	How would you rate the overall palliative care clinic services?	93 (84%)	3 (3%)	10 (9%)	3 (3%)	1 (1%)

## Discussion

Palliative care is the branch of medicine that focuses on patients with chronic life-threatening illnesses [4]. Cancer falls under the umbrella of palliative care, as well as a broad spectrum of long-term health problems that eventually will interfere with patients' well-being and life expectancy. Heart failure, chronic obstructive pulmonary disease, Alzheimer's disease, acquired immunodeficiency syndrome (AIDS), and many other health problems are under the scope of palliative care. Even though palliative care is a relatively new subspecialty in medicine, it has become well recognized and advocated by the WHO as a basic human right. Nevertheless, 40 million people worldwide need palliative care services. This includes 5.5 million patients with terminal cancer and one million patients with end-stage AIDS, many of whom endure moderate to severe pain without having access to pain medications such as morphine, although morphine is not an expensive drug and costs only a few cents per unit [3]. The main goals of palliative care are to alleviate the suffering caused by the chronic illness and to help patients and families cope during the difficult times as the disease progresses. To enhance the quality of life, a holistic multidisciplinary framework is adopted in palliative care promotion. Palliative care deals with the patient's suffering through a comprehensive approach, including all possible impacts of a given disease, and seeks to deliver reasonable symptom control, relief, and achievement of a better outcome. It targets not only the physical symptoms but also the psychological, social, and spiritual issues that might complicate a chronic disease.

Palliative care in Saudi Arabia

Palliative care services were first introduced in Saudi Arabia more than 20 years ago at King Faisal Specialist Hospital and Research Center in Riyadh [2]. Since then, the field has grown gradually. Now there are several secondary and tertiary care hospitals that provide palliative care service through inpatient units and consultation services, as well as outpatient clinics and home care teams. However, the cancer rate in Saudi Arabia is expected to increase over the next few years due to demographic changes [2]. Thus, the need for palliative care service expansion in Saudi Arabia is fundamental. The scope of palliative care service includes providing ongoing assessment and management of pain and other distressing symptoms [1,4]. The ability to identify primary concerns and perform a comprehensive assessment for patient symptoms to manage them appropriately is an essential part of the palliative care practice. A valid tool for symptom assessment is vital; therefore, any training program should focus on this area [2]. Palliative care asserts life and addresses dying as a natural process; nevertheless, it never aims to expedite or stall death. Palliative care deals with the symptomatic part of a given disease to ensure that the patient can live with the best possible integrity. Death eventually will come, but it should be perceived within its natural context. Palliative care service should not interfere with hastening nor stalling it [3]. Palliative care offers a support system to help patients live as actively as possible until the time of their natural death. As mentioned before, the palliative role is to boost the quality of life to the best expectation of the patient and family by utilizing all available facilities [4]. Palliative care supports the family throughout the disease and during bereavement. Providing care for patients with chronic terminal diseases is a distressing task for immediate family members and caregivers. They have a genuine need for a combined support system throughout the disease progression and after the patient's death [5]. Palliative care is also applicable early in the disease course, in alignment with other curative therapies such as chemotherapy and radiation. It is now an evidence-based practice to incorporate palliative care service early in the disease course (i.e., soon after a diagnosis is established), as it has been found to have a profound impact on the outcome later in the course of the disease and toward the end of life [4,6]. Continuous education and training for the palliative care staff are essential to help them maintain their competence and stay aware of the developing areas of their field [1,5]. The healthcare sector in Saudi Arabia is undergoing a tremendous positive transformation as part of the vision 2030, Model of Care (MOC) project. In order to achieve this transformation-and in conjunction with the WHO recommendations-it is essential to implement palliative care service at the level of primary healthcare and to provide palliative care services early in the disease course and ensure accessibility to the target population. One hindrance to this goal is the limited number of well-trained providers who can accommodate current and future needs in the country and ensure patient care and satisfaction [6].

Patient waiting time depends on many factors, such as the doctor's specialty, the kind of patients he or she consults, the area where he or she practices, and the efficacy and availability of supportive staff. The amount of time a patient spends in the emergency department or outpatient clinic plays a significant role in determining their satisfaction. With the increase in medical care facilities and advanced medical technology, there are many choices available. Therefore, few people will return to see a doctor who has no respect for their time [7]. Also, patient satisfaction helps maintain an organization's reputation, and in return, transforms into improving healthcare service and patient trust [8,9].

**Figure 1:**
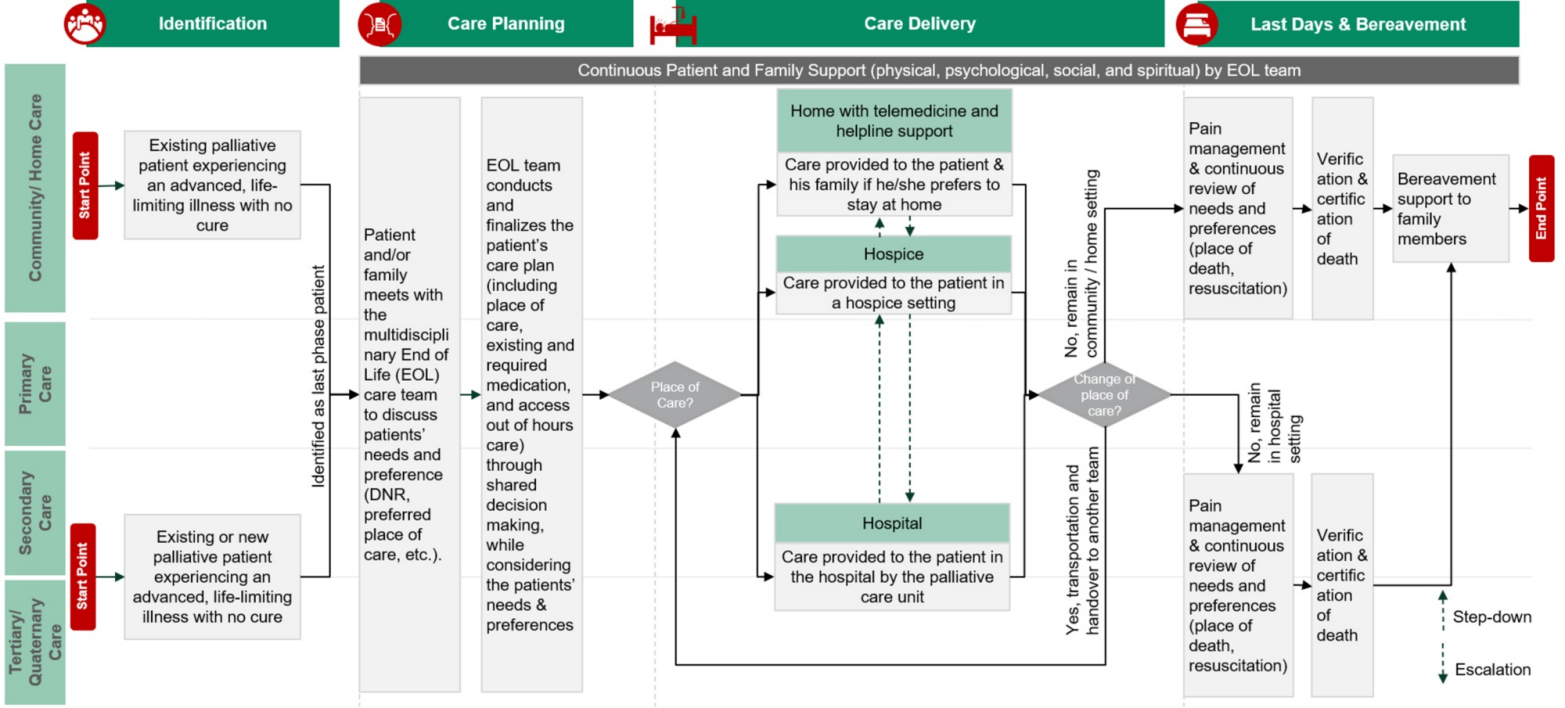
High-level last phase pathway map Last Phase Pathway* focuses on improving the quality of life of patients and their caregivers experiencing an advanced, life-limiting illness. *Pathway that focuses on improving the quality of life of patients and their families facing life-threatening illness, through the prevention and relief of suffering by means of early identification, targeted care planning and treatment of pain in its physical, psychosocial and spiritual forms. Abbreviations: EOL, end of life; DNR, do not resuscitate.

Inadequate information is a contributing factor to a cancer survivor's psychological state, anxiety, or depression [10-12]. Providing clear and adequate information on patient health and treatment is a key factor for palliative care for cancer patients during follow-up care and to improve their quality of life [10]. A previous study revealed that cancer patients who received information about potential late effects of their cancer therapy, as well as risk-based screening recommendations, had positive reactions to this information, and their tension and anxiety did not increase (Figure 1) [12].

This study revealed that the integration of palliative care into primary healthcare has a positive impact on patients. Clinic waiting time is one of the determinants of patient satisfaction worldwide, although it depends mainly on the availability of healthcare providers and the structure of the institutions. Previous studies revealed waiting times ranging from 60 to 180 minutes and patient satisfaction of 89.3% [5,7], which supports our findings from our participants who attended PHCCs. Waiting time was satisfactory to our patients; patients spent less time waiting to be seen by the doctor in the primary health center compared to a secondary/tertiary health center because of the shorter waiting times at the PHCC. Therefore, assessing patient satisfaction can help improve and retain the quality of service delivery in primary healthcare. The information given was appropriate, and 89% of patients were extremely satisfied. This corresponds to the previous study patients who received information about their cancer therapy, potential late effects, and risk-based screening recommendations-their feedback was positive, and their tension and anxiety did not increase [12]. This was an indicator of the efficiency of the education given to our patients at the primary healthcare level.

Study limitations

Our study was limited in that it was a description of a single centre experience in integrating palliative care into primary care, and our sample size was small. A similar study done on the national level will reveal newer challenges that may require newer and improved strategies to address.

## Conclusions

The integration of palliative care into primary healthcare increases the patient quality of life and patient satisfaction. Integration of palliative care into primary healthcare is more beneficial for patients with advanced cancer than palliative care consultations offered on-demand from referral healthcare institutions. Additionally, palliative care improved patient adherence and compliance with cancer treatment and improved quality of life and patient satisfaction through education. This model can be generalized in all PHCCs in Saudi Arabia.

## References

[1] (2003, Geneva). World Health Organization. The World Health Report 2002: reducing risks, promoting healthy life.

[2] Alshammary S, Duraisamy B, Albalawi Y, Ratnapalan S (2019). Development of palliative and end of life care: the current situation in Saudi Arabia. Cureus.

[3] (2020). World Health Organization (WHO). Fact sheet N 402, palliative care. https://www.who.int/news-room/fact-sheets/detail/palliative-care.

[4] (2020). World Health Organization (WHO). WHO definition of palliative care. https://www.who.int/cancer/palliative/definition/en/.

[5] Vanbutsele G, Pardon K, Van Belle S (2018). Effect of early and systematic integration of palliative care in patients with advanced cancer: a randomised controlled trial. Lancet Oncol.

[6] (2020). California Health Care Foundation: Wavering palliative care into primary care: a guide for community health centers. https://www.chcf.org/wp-content/uploads/2017/12/PDF-WeavingPalliativeCarePrimaryCare.pdf.

[7] Prakash B (2010). Patient satisfaction. J Cutan Aesthet Surg.

[8] Geberu DM, Biks GA, Gebremedhin T, Mekonnen TH (2019). Factors of patient satisfaction in adult outpatient departments of private wing and regular services in public hospitals of Addis Ababa, Ethiopia: a comparative cross-sectional study. BMC Health Serv Res.

[9] Andaleeb SS (1998). Determinants of customer satisfaction with hospitals: a managerial model. Int J Health Care Qual Assur.

[10] van de Poll-Franse LV, Nicolaije KA, Ezendam NP (2017). The impact of cancer survivorship care plans on patient and health care provider outcomes: a current perspective. Acta Oncol.

[11] Mesters I, van den Borne B, De Boer M, Pruyn J (2001). Measuring information needs among cancer patients. Patient Educ Couns.

[12] Oeffinger KC, Hudson MM, Mertens AC (2011). Increasing rates of breast cancer and cardiac surveillance among high-risk survivors of childhood Hodgkin lymphoma following a mailed, one-page survivorship care plan. Pediatr Blood Cancer.

